# Update of the Dutch manual for costing studies in health care

**DOI:** 10.1371/journal.pone.0187477

**Published:** 2017-11-09

**Authors:** Tim A. Kanters, Clazien A. M. Bouwmans, Naomi van der Linden, Siok Swan Tan, Leona Hakkaart-van Roijen

**Affiliations:** 1 Institute for Medical Technology Assessment, Erasmus School of Health Policy & Management, Erasmus University Rotterdam, Rotterdam, the Netherlands; 2 Centre for Health Economics Research and Evaluation, University of Technology Sydney, Sydney, Australia; 3 Erasmus MC University Medical Center, department of Public Health, Rotterdam, the Netherlands; Post Graduate Institute of Medical Education and Research School of Public Health, INDIA

## Abstract

**Objectives:**

Dutch health economic guidelines include a costing manual, which describes preferred research methodology for costing studies and reference prices to ensure high quality studies and comparability between study outcomes. This paper describes the most important revisions of the costing manual compared to the previous version.

**Methods:**

An online survey was sent out to potential users of the costing manual to identify topics for improvement. The costing manual was aligned with contemporary health economic guidelines. All methodology sections and parameter values needed for costing studies, particularly reference prices, were updated. An expert panel of health economists was consulted several times during the review process. The revised manual was reviewed by two members of the expert panel and by reviewers of the Dutch Health Care Institute.

**Results:**

The majority of survey respondents was satisfied with content and usability of the existing costing manual. Respondents recommended updating reference prices and adding some particular commonly needed reference prices. Costs categories were adjusted to the international standard: 1) costs within the health care sector; 2) patient and family costs; and 3) costs in other sectors. Reference prices were updated to reflect 2014 values. The methodology chapter was rewritten to match the requirements of the costing manual and preferences of the users. Reference prices for nursing days of specific wards, for diagnostic procedures and nurse practitioners were added.

**Conclusions:**

The usability of the costing manual was increased and parameter values were updated. The costing manual became integrated in the new health economic guidelines.

## Introduction

Economic evaluations in health care are increasingly used to inform decision makers on value for money of health care interventions. Standardization of methodology for economic evaluations is needed to ensure high-quality evaluations and obtain outcomes that can be compared between health care interventions. For this purpose, pharmacoeconomic guidelines have been developed in various countries [1]. These guidelines differ between countries, for example with respect to which costs should be included, methodology of calculating costs and discounting.

In 2015, The Dutch National Health Care Institute (ZIN) updated the pharmacoeconomic guidelines to align formerly co-existing health economic guidelines in the Netherlands, connect with guidelines in other countries and expand guidelines to non-pharmaceutical interventions. The guidelines prescribe a societal perspective, meaning all relevant costs, should be included in an economic evaluation. The guidelines and accompanying modules were published in February 2016 [2,3]. The guidelines are accompanied by several ‘modules’, among them a module for costing studies (hereafter the ‘costing manual’).

The purpose of the costing manual is to provide guidance to researchers and policy makers to perform and evaluate economic evaluations of health care interventions. The first Dutch costing manual was published in 2000. Since then, two updates were published in 2004 and 2010 [4]. The instrument has been widely used since the publication of the first costing manual. Standard cost prices for various health care services, called reference prices, constitute an important part of the costing manual. Regular updates of the costing manual are essential in order to reflect changes in health care, price increases, and developments in HTA research. This paper reports on the update of the Dutch costing manual which serves the purpose of harmonizing the costing side of economic evaluations in health care. As the costing manual is written in Dutch, this paper can provide international readers with a better understanding of the content and methodologies used in the costing manual and provide them with the reference prices for healthcare services for the Netherlands.

## Materials and methods

The revision of the costing manual was part of the new edition of the Dutch health economic guidelines. The principles of the costing manual had to be aligned with these guidelines and the reference prices were to be updated. The update of the costing manual consisted of three separate steps. First, an inventory of user needs was made. Second, the content of the manual was updated to resemble the health economic guidelines and adjust methodological paragraphs. Furthermore, reference prices were updated through literature and database research and stakeholder consultation. Third, members of the expert committee supervising the guideline revision for ZIN were asked to comment on intermediate and final results of the revision of the costing manual. The revision of the costing manual was supervised by a team of ZIN with experience in health economics and project management.

### Inventory of user needs

An online survey was sent out to over 700 people (mainly from universities, industry, governmental bodies, health care institutions and consultancy) from the target audience of the costing manual to investigate user satisfaction with the previous costing manual, and to identify user needs and opportunities for improvement for the updated costing manual. The online survey consisted of 13 questions, and encompassed three themes: clarity and user-friendliness of the previous manual; methodological issues; and (need for additional) reference prices. The online survey opened on January 15^th^ 2015 and closed on February 2^nd^ 2015. During this period, one reminder was sent out to non-respondents. The survey questions are provided in S1 Appendix.

### Update of the costing manual

Changes to the content of the costing manual that were made to align with the new health economic guidelines included incorporating a new typology of costs and consequently updating the roadmap for costing studies. The roadmap describes the steps that are needed to conduct a costing study [4]. It serves as a starting point for conducting costing studies and connects the health economic guidelines to the costing manual.

Reference prices for health care consumption, which are average unit costs, constitute a frequently used part of the costing manual. Reference prices were recalculated using recent information on costs, volume and prices for various types of health care services. Reference prices were updated using various techniques (summarized in Table 1), depending on data availability. If possible, bottom-up microcosting was used to calculate reference prices, as this is the gold standard for calculating cost prices [5]. When bottom-up microcosting data was not available, grosscosting methods were applied to calculate reference prices. Bottom-up microcosting studies, identifying and valuating resource use per individual patient, were used to calculate references prices for hospital care [Tan, S.S., et al. *Reference unit prices for surgery*, *neurology and paediatrics*. Submitted for publication]. Reference prices for emergency care, ambulances, blood products, daycare treatment in mental health care and rehabilitation were calculated using top-down grosscosting, for which data on costs and volumes were derived from health care providers. Data on expenditures and volumes derived from national health care database were used to calculate reference prices using top-down grosscosting, for primary care physicians, paramedical care, elderly care, home care, mental health care and health care for disabled patients [6]. Finally, tariffs were used to value diagnostic procedures [7]. For contacts with independent psychotherapists and psychiatrists, ambulatory consultation in a general institution and inpatients days in mental health care tariffs were used [8]. Relevant stakeholders were consulted to validate the updated reference prices. Updated informal care costs were derived from the website of the Central Administration Office (CAK). Productivity costs should be valued using the friction cost method based on the Dutch health economic guidelines. The friction period is equal to the average duration of a job vacancy plus an additional four weeks. The average duration of job vacancies was calculated with the following formula: 365 / (the number of filled vacancies in one year / the number of vacancies at a moment in that same year). The number of vacancies was derived from the website of Statistics Netherlands. Wage levels were also derived from the Statistics Netherlands website.

**Table 1:** Sources used to calculate reference prices for various health care services.

Health care service	Sources for reference prices
Hospital days	Bottom-up costing studies
Outpatient visits	Bottom-up costing studies
Emergency room visit	Health care providers
Ambulance transport	Health care providers
Primary care physician	National health care database
Paramedical care	National health care database
Elderly care	National health care database
Home care	National health care database
Mental health care	National health care database, health care providers, tariffs
Rehabilitation therapy	Health care providers
Health care for disabled patients	National health care database
Informal care	Central Administration Office
Friction period	Statistics Netherlands
Wage rates	Statistics Netherlands

### Expert committee

An expert committee of 12 experienced health economists supervised the revision of the Dutch health economic guidelines and were also asked to provide input to the costing manual during the research period (January 2015 through June 2015). A draft report of the costing manual was peer-reviewed by two members of the expert committee.

## Results

### Survey results

A total of 71 respondents completed the survey (non-response of about 90%). Respondents were predominantly employed at universities and health care providers. Most respondents agreed with the statements that the costing manual was user friendly (75%); clear (75%) and well-written (76%). These outcomes were discussed with ZIN and the expert committee. Although no pre-specified thresholds were used for these questions, the results were judged to be sufficient. Respondents indicated that their main use of the costing manual was to obtain reference prices; 86% of respondents stated to use the reference prices in costing studies or economic evaluations. Reference prices not being available for health care services that were investigated was the main reason for not using reference prices. Some respondents (n = 3) stated that reference prices were outdated and two respondents questioned the reliability of the reference prices. Alternative sources that were used by respondents to value health care services were tariffs, DRG prices, empirical research, financial administrations and expert panels. The relevance of the responses to the survey was judged by the authors, and discussed with ZIN and the external expert committee. Respondents identified a number of potential improvements for the costing manual. First, reference prices needed to be updated. Second, reference prices for the previously ignored categories ‘nurse practitioner’ and ‘diagnostic procedures’ were requested. These were the only health care services for which reference prices were requested by more than two respondents. Furthermore, respondents claimed that the methodological description of calculating productivity losses and costs for medication use could be improved.

### Adaptations to the costing manual

In line with the revised health economic guidelines, in which the typology of costs was changed to reflect the classification used in the Drummond textbook [9]; 1) costs within the health care sector; 2) patient and family costs; and 3) costs in other sectors. Future medical costs, which was a separate category in the taxonomy used in the previous costing manual, are included in the category ‘cost within the health care sector’.

**Fig 1:**
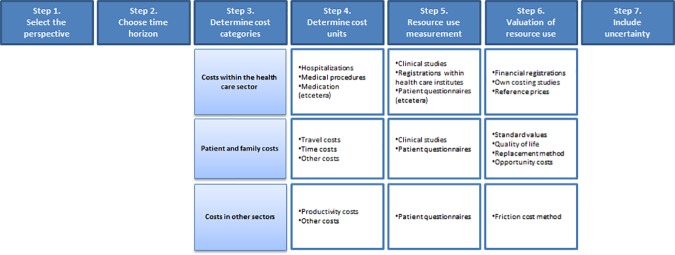
Roadmap for costing studies.

In the updated costing manual, the roadmap for costing studies was adjusted to reflect the new classification of costs. The revised roadmap is provided in Fig 1 and comprises the following seven steps: 1) select the perspective; 2) adopt an appropriate time horizon; 3) determine cost categories; 4) determine cost units; 5) measure resource use; 6) value resource use; and finally 7) include uncertainty around the estimates.

#### Costs within the health care sector

The costing manual identifies the reference prices to be the most appropriate to value costs within the health care sector. Table 2 provides the reference prices for the most important health care services.

**Table 2:** Reference prices for costs within the health care sector.

Health care service	Reference price ^a^
Inpatient hospital day (including materials, equipment, etc.)	
- General hospital	€ 443
- University hospital	€ 642
- Weighted average	€ 476
Medical specialty	
- Surgery	€ 405
- Neurology	€ 395
- Paediatrics	€ 627
- Haematology-oncology	€ 636
Intensive care unit day	€ 2015
Daycare treatment	€ 276
Outpatient visit (including materials, equipment, etc.)	
- General hospital	€ 80
- University hospital	€ 163
- Weighted average	€ 91
Medical specialty	
- Surgery	€ 73
- Neurology	€ 99
- Paediatrics	€ 101
- Haematology-oncology	€ 132
Emergency room visit	€ 259
Primary care physician/General practitioner	€ 33
Paramedical care (per visit)	
- Physical therapy	€ 33
- Exercise therapy	€ 34
- Speech therapy	€ 30
- Occupational therapy	€ 33
Elderly care	
- Inpatient elderly care incl. daycare, per day	€ 168
- Daycare	€ 67
Home care (per hour)	
- Household activities	€ 20
- Personal care at home	€ 50
- Support at home	€ 58
- Nursing at home	€ 73
- Home treatment	€ 120
Mental health care (per visit)	
- Primary care physician	€ 66
- Nurse practitioner	€ 17
- Social worker	€ 65
- Primary care psychologist	€ 64
- Independent psychotherapist	€ 94
- Independent psychiatrist	€ 94
- Ambulatory consultation general institution	€ 98
- Inpatient day	€ 302
- Daycare treatment	€ 169
Rehabilitation therapy	
- Rehabilitation therapy per hour	€ 153
- Daycare treatment (children)	€ 521
- Daycare treatment (adults)	€ 460
Health care for disabled patients	
- Inpatient care for mentally disabled patients incl. daycare, per day	€ 209
- Inpatient care for physically disabled patients incl. daycare, per day	€ 205
- Inpatient care for aurally disabled patients incl. daycare, per day	€ 310
- Inpatient care for visually disabled patients incl. daycare, per day	€ 217

^a^ Sources and calculations are provided in the costing manual [13]

An important refinement in the updated costing manual was the specification of unit prices for hospital days and outpatient visits according to medical specialty, next to generic references prices for hospital days and outpatient visits. Reference prices for hospital days ranged from €395 at the neurology department, to €636 for a day at the hematology-oncology department. Reference prices for outpatient visits ranged from €73 (surgery department) to €132 (hematology-oncology department).

The methodology for calculating medication costs was simplified. Previously, a claw-back had to be included in calculating medication costs, but due to changes in legislation the claw-back has ceased to exist. The calculation of medication costs, currently consists of two components: 1) the price of the medication itself (including VAT), which can be derived from www.medicijnkosten.nl and 2) delivery costs of the pharmacy, which is €6.00 for regular deliveries.

In response to the survey outcomes, the updated version includes tariffs for the most common diagnostic procedures, such as common laboratory assessments, MRI and CT-scan. Generally, these tariffs come close to the actual costs. Tariffs for other diagnostic procedures can be found on the website of the Dutch National health care authority (NZa) and are updated regularly.

Furthermore, a reference price for nurse practitioner in mental health care was included upon request of the survey respondents. Furthermore, the costing manual was supplemented with reference prices for health care services for disabled patients.

Future medical costs related to the disease of interest can be assessed as current costs within the health care sector. In contrast, future medical costs for unrelated diseases cannot be assessed in the same way, as it cannot be predicted which unrelated diseases a patient will suffer from in the future. Therefore, future medical costs for unrelated diseases have to be calculated on the basis of average health care usage per person, rather than calculating costs per patient. The Practical Application to Include future Disease costs (PAID) can be used to do this [10]. In PAID, double counting of related future medical costs is prevented, as the user can identify which diseases are already included in the costing study. PAID corrects for the observation that health care costs increase with age and are highest in the last year of life. PAID is available online through www.imta.nl/paid.

#### Patient and family costs

The value of time on informal care is an important aspect of patient and family costs and should be included in costing studies according to the Dutch health economic guidelines. Data on the volume of informal care can be obtained by means of diaries or questionnaires. An updated reference price for informal care was calculated using the opportunity cost method [11] and was determined to be €14 per hour. Another component of patient and family costs are travel costs, for which parameter values were also updated. The average distance to a hospital in the Netherlands was 7.0 kilometres; the average distance to a GP was 1.1 kilometres. Costs per kilometre were €0.19 by public transport or by car (excluding parking costs of €3.00 per visit).

#### Costs in other sectors

The third type of costs in the Drummond taxonomy are costs in other sectors. An important component of costs in other sectors is costs due to lost productivity. The Dutch health economic guidelines prescribe the inclusion of productivity losses in economic evaluations. Productivity costs should be calculated using the friction cost method, which assumes that productivity costs are only incurred during the period between the moment an employee falls ill and the moment the employee is replaced, the so-called friction period [12]. All parameters needed to calculate productivity losses with the friction cost method were updated; the friction period was estimated to be 85 days and average wage rates were updated, equalling €34.75 per hour. When an intervention is solely focussed on men or women, gender-specific wage rates can be used (€37.90 per hour for men and €31.60 per hour for women). In response to survey respondents’ requests, the description of methodology to calculate productivity losses was improved. Three examples were included to provide hands-on information on how to calculate productivity costs.

### Dissemination of costing manual and reference prices

Along with the updated costing manual, a Microsoft Excel instrument was developed that contained all updated reference prices and parameters in the costing manual. The instrument will be hosted by ZIN and enables users to quickly find reference prices. Furthermore, users can select a reference year, resulting in inflation-corrected reference prices.

## Discussion

The costing manual is an essential part of the revised Dutch health economic guidelines, which were published in February 2016. The costing manual describes the methodology of costing studies and reference prices, which are used to increase the quality and comparability of costing studies. As such, the costing manual is a widely used instrument for costing studies and economic evaluations in health care in the Netherlands. This paper describes the updates in the revised version of the Dutch costing manual. Important revisions were simplification of methodology and updates of reference prices, including those for a number of hospital specialties and diagnostic procedures. The updated costing manual is freely available from the website from ZIN (www.zorginstituutnederland.nl) together with an online Microsoft Excel instrument containing the reference prices to ensure accessibility (available through www.imta.nl/costingtool).

Standardization of methodology for health economic studies enables comparison of studies’ outcomes within countries. Internationally, differences in health economic guidelines remain [1]. One particular factor that differs between national guidelines concerns the perspective of economic evaluations, i.e. whether studies should adopt a health care perspective or a wider, societal perspective [14]. Even in countries that adopt a societal perspective and include productivity costs, the methodology to calculate productivity costs can differ–the Netherlands is one of the few countries that applies the friction cost method [4]. The detailed description of this methodology in the costing manual, including examples, is therefore essential for a good understanding of this approach. The iMTA productivity cost questionnaire (iPCQ) can be used to quantify productivity losses, and enables calculation of productivity costs according to the friction cost method [15]. The accompanying manual provides a step by step explanation on measuring and valuing productivity losses [16].

Furthermore, adopting a societal perspective also entails including costs of informal care. Research has shown that including informal care costs can influence the outcomes of a cost-effectiveness study considerably [17]. The use of a single reference price as provided in the costing manual is therefore essential, as the use of informal care is increasing [18].

Health economic guidelines prescribe the use of country-specific unit prices, to reflect absolute and relative differences in unit prices [19]. However, a recent study found that a standard cost list, such as the reference prices provided in the costing manual, is only available in four out of 30 pharmacoeconomic guidelines [20]. Use of a costing manual and reference prices ensures that differences in costs result from differences in health care utilization and not from the methodology applied to calculate costs. The absence of standard prices leads to differences in valuation of the same health care service within a single country, and can influence study outcomes and potentially even reimbursement decisions. Next to reference prices published in the costing manual, the manual provides guidance on the methodology of calculating unit prices when reference prices are not available. As such, using standard methodology increases the comparability and transparency of unit prices used in costing studies. In this way, the Dutch costing manual can be a useful tool for developing costing manuals in other countries.

### Limitations

This paper provides reference prices for the most important health care services. The use of reference prices increases the comparability between studies. However, a balance between standardization and the specific situation of an economic evaluation has to be found. Differences in costs between providers or patients are not accounted for in reference prices. For those economic evaluations in which one particular type of health care has a large impact on the results, researchers should therefore ensure that reference prices reflect the costs of the situation specific to their study.

For diagnostic procedures, reference prices were not available. Tariffs were considered to be an accurate estimate of actual costs of diagnostic procedures. Mental health care covers different echelons, and ranges from contacts with mental health nurse practitioners to inpatient days. Some, but not all, estimates of the various reference prices for mental care were based on tariffs. For instance, reference prices for contacts with mental health nurse practitioners, primary care psychologists and primary care physician were based on the total costs divided by the total number of contacts/days. In case we necessarily had to base the estimates on tariffs, we used charges for contacts or hospital days not reimbursed by public health care. These are the charges clients have to pay if they are not covered under the Dutch Healthcare Insurance Act. These tariffs are assumed to reflect the actual costs in the best possible way, as these are calculated using the actual input of personnel and resources. Unfortunately, the researchers did not have access to the calculations and hence were not able to recalculate these estimates. For now, face validity seems to verify these reference prices, but further research is recommended.

The requested reference price for a nurse practitioner could only be provided for nurse practitioners in mental health care, as the available data did not allow to estimate a reference price for general nurse practitioners in a GP setting. Bottom-up research is needed to determine the reference price for general nurse practitioners in this setting.

Bottom-up costing studies are considered the gold standard for calculating cost prices [5]. However, this method is time consuming and costly. Therefore, due to data availability, reference prices in the costing manual could not be based solely on bottom-up costing studies. When additional bottom-up costing studies will be performed in the future, the resulting prices might replace existing reference prices in future updates of the costing manual.

The response rate of the online survey was approximately 10%. However, as the purpose of the survey was identification rather than quantification of user needs, the low response rate was not considered problematic.

### Future updates of the costing manual

The values in the costing manual should be regularly updated, to ensure that methodology reflects current best practice and reference prices reflect current price levels. Future updates also entail including additional reference prices for other types of health care and more detailed reference prices, for instance for other hospital specialties. The expert committee proposed that one way to increase the availability of reference prices is to set up an open repository, in which researchers can share unit prices derived in their own studies. However, a number of questions need to be answered before such a repository could be established, such as, who would be responsible for hosting the repository, who should finance the repository, what requirements should be used to assess the quality of reference prices and who should be responsible for checking the quality of the reference prices. In this respect, the Excel tool is particularly useful, as this instrument can be updated more quickly than a hard-copy of the costing manual. An upcoming English translation of the online instrument also enables foreign users to easily access Dutch reference prices, and consequently further increase transparency and comparability of Dutch costing studies in health care.

## Supporting information

S1 Appendix Survey: Input update costing manual 2015. (DOCX)
