# Supplementary material for: Update of the Dutch manual for costing studies in health care
Source: PLoS One. 2017 Nov 9;12(11):e0187477. doi: 10.1371/journal.pone.0187477 (PMC5679627; doi:10.1371/journal.pone.0187477)
Supplement: S1 Appendix — (DOCX) [file pone.0187477.s001.docx]

1. What type of organization do you work for?
   - Consultancy
   - Pharmaceutical industry
   - Government
   - University
   - Health care provider
   - Other, please specify
2. Are you involved in economic evaluations/costing studies
   - No
   - Yes, for scientific research
   - Yes, for reimbursement decisions
   - Yes, for other reasons
3. The following statements are about the description of methods of costing studies in the costing manual, which were drafted following remarks raised by previous users. Please indicate if you agree with these statements (1=strongly disagree; 4=strongly agree).
   1. I think that the action plan is clear
   2. I think that the description for calculating medication costs is clear
   3. I think that the description for adapting reference prices is clear
   4. I think that the description of calculating productivity costs is clear
4. If you have additional remarks concerning the methodology in the costing manual, please state them here
5. Which datasources do you use for costing studies?
   - Reference prices
   - Financial administrations
   - Tariffs
   - DRGs
   - Empiric research
   - Expert panels
   - Other sources, please specify …
6. If you have additional remarks regarding the datasources, please state them here
7. What is the reason for not using the reference prices from the costing manual (if applicable)?
   - Reference prices are outdated
   - Reference prices are not available for specific health care providers
   - Reference prices are not valid
   - Other reasons, please specify …
8. Do you feel that reference prices are missing for certain health care providers?
   - No
   - Yes, namely for the following health care providers …
9. The following statements are about the format of the costing manual. Please indicate if you agree with these statements (1=strongly disagree; 4=strongly agree).
   1. I think that the manual is user friendly
   2. I think that the manual is clear
   3. I think that the manual is clearly written
   4. An interactive version of the manual would be useful
10. If you have additional remarks concerning the format of the manual, please state them here
11. Do you think that improvements to the costing manual are necessary with regard to the following aspects:
    1. Description methodology, namely …
    2. Validation reference prices, namely …
    3. Description reference prices, namely …
    4. Other aspects, namely …
12. In addition to the usual costing manual, an online version with reference prices will be developed. What information would you like to see included in this digital version?
13. If you any other remarks with regard to improving the costing manual, please state them here (e.g. points of improvements, any unclear issues, missing issues)
